# Uncovering the Neglected Floral Secretory Structures of *Rhamnaceae* and Their Functional and Systematic Significance

**DOI:** 10.3390/plants10040736

**Published:** 2021-04-09

**Authors:** Carimi Ribeiro, Cristina Marinho, Simone Teixeira

**Affiliations:** 1 Post-Graduate Program in Comparative Biology, Faculdade de Filosofia, Ciências e Letras de Ribeirão Preto, Universidade de São Paulo, 14040-901 Ribeirão Preto, Brazil; carimicortez@hotmail.com; 2 Faculdade de Ciências Farmacêuticas de Ribeirão Preto, Universidade de São Paulo, 14040-903 Ribeirão Preto, Brazil; 3 Real Jardín Botánico, CSIC, 28014 Madrid, Spain; crm.botanica@gmail.com

**Keywords:** cavities, colleters, ducts, idioblasts, glands, mucilage, tannins, Rhamnoid, Rosales, Ziziphoid

## Abstract

Rhamnaceae flowers are notably recognized by their fleshy nectary. Other types of floral secretory structures have been scarcely reported for this family. Thus, the objective of the present study was to update the occurrence of these structures in the family and to contribute to the knowledge of their morphology and systematic significance. To this end, we carried out an extensive bibliographic search on the secretory structures of the family and obtained data for 257 taxa. Additionally, we presented here novel data (surface, anatomy, and ultrastructure) for six species belonging to the main clades within Rhamnaceae. The family has a wide diversity of types of mucilage-secreting structures: epidermis, hypodermis, idioblasts, cavities, and ducts. Mucilage and phenolic idioblasts are widely distributed among the floral organs. Colleters are present in all sampled species, and these are the first reports of their occurrence in floral organs of Rhamnaceae. The information obtained about the structure, secreted content, and occurrence of the secretory structures of Rhamnaceae helped us to understand the assertive folk use of its species. The absence of mucilage and the presence of resin or mucilage cavities and ducts in some taxa may have intrafamily systematic significance.

## 1. Introduction

Secretory structures not only play an important role in the eco-physiological relationships of plants with the environment but frequently constitute relevant traits to untangle the phylogenetic relationships between lineages within angiosperm families. Rhamnaceae, a family belonging in the order Rosales, that comprises about 55 genera and 900 species, is notably recognized by the presence of showy floral nectaries [1,2]. Due to its easy visualization, this type of floral secretory structure has been the most studied in the group, both in terms of its functionality [3,4,5,6,7] and morphology [8,9]. Likewise, this family is also recognized by the presence of epidermal cells, idioblasts, and cavities/ducts that secrete mucilage, found in its vegetative organs, mainly leaves [10,11,12,13,14,15,16,17,18,19,20,21,22]. However, morphological studies of other floral secretory structures of Rhamnaceae are scarce. Previously, these lesser-known structures had been reported only by Schirarend and Hoffmann [23], who explored the floral anatomy of 18 species of the genus *Reynosia* and in a study recently published by Gotelli et al. [24], which describe and compare the structure of the osmophores in 11 species of the main clades of the family.

Apart from its morphological diversity, many studies in the family are related to their traditional uses. A wide range of Rhamnaceae species are popularly used as medicines by several human communities [25]. Their pharmacological uses in folk medicine are due to their curative properties, which are provided by products of secondary metabolism. It is notable that most research in the family has been concentrated on screening the chemical compounds and the biological activities of plant extracts of specific genera such as *Ziziphus* (*Z. jujuba*, *Z. joazeiro*, *Z. spina-christi*) and *Hovenia* (*H. dulcis*) due to the commercialization of their fruits and pseudo-fruits [17,25,26,27,28,29,30,31,32,33,34,35,36,37]. This type of interest restricts the sampling in relation to the total number of species. Recently, the use of rhamnose in anti-aging facial moisturizers has introduced the family in the interest of the cosmetic industry. Rhamnose is mainly found in the leaves of the *Rhamnus* species and has also been reported in species of *Ziziphus.* This monosaccharide, together with glucose and galactose, are the main components of the mucilage found in these species, which have been connected to water stress tolerance [17].

Secretory structures vary enormously in complexity and can be classified according to their secretions (oil, resins, phenolic compounds, mucilage, gums, lattices, etc.), structure (cavities, ducts, idioblasts, nectaries, osmophores, elaiophores, trichomes, and others), and origin (trichome, emergence) [38,39]. Because of its great diversity, certain types of secretory structures can be taxonomic trademarks for some botanical groups, e.g., laticifers in Euphorbiaceae [40], Moraceae [41] and Apocynaceae [42], peltate glandular trichomes in Lamiaceae [43], secretory cavities in Myrtaceae [44], and oil idioblasts in Lauraceae [38], among others. For Rhamnaceae, some works published between the late nineteenth and early twentieth centuries [10,11,12] demonstrate that some secretory structures may play a diagnostic role in the family. There is also an attempt to associate the types of floral nectary (indistinct, adpressed, annular, revolute) with the group’s systematic [8,9].

Thus, the objective of the present study was to show that Rhamnaceae has a huge diversity of floral secretory structures other than nectaries and, consequently, a great potential for further and diverse studies. In addition, we intended to check if the type and location of the secretory structures of the family represent a systematic value for the group. To achieve this goal, we carried out an extensive bibliographic review on the secretory structures of the family, dating back from the end of the nineteenth century to the present day. Additionally, we investigated novel data for six species of five genera belonging to the main clades within Rhamnaceae, Ziziphoid, and Rhamnoid *sensu* Hauenschild et al. [45]. The data obtained are discussed in terms of ecological interactions and the establishment of synapomorphies for the family and for the remaining members of the order Rosales. The information presented here helped us to reassess the great systematic potential of Rhamnaceae secretory structures and the assertive folk use of its species.

## 2. Results

### 2.1. Novel Data

The six studied species of Rhamnaceae exhibited different types of floral secretory structures other than the diagnostic nectaries: colleters (all species), mucilaginous and phenolic idioblasts (all species), and oil-resin cavities and ducts (*R. elaeocarpum*) (Table 1).

**Figure 1:**
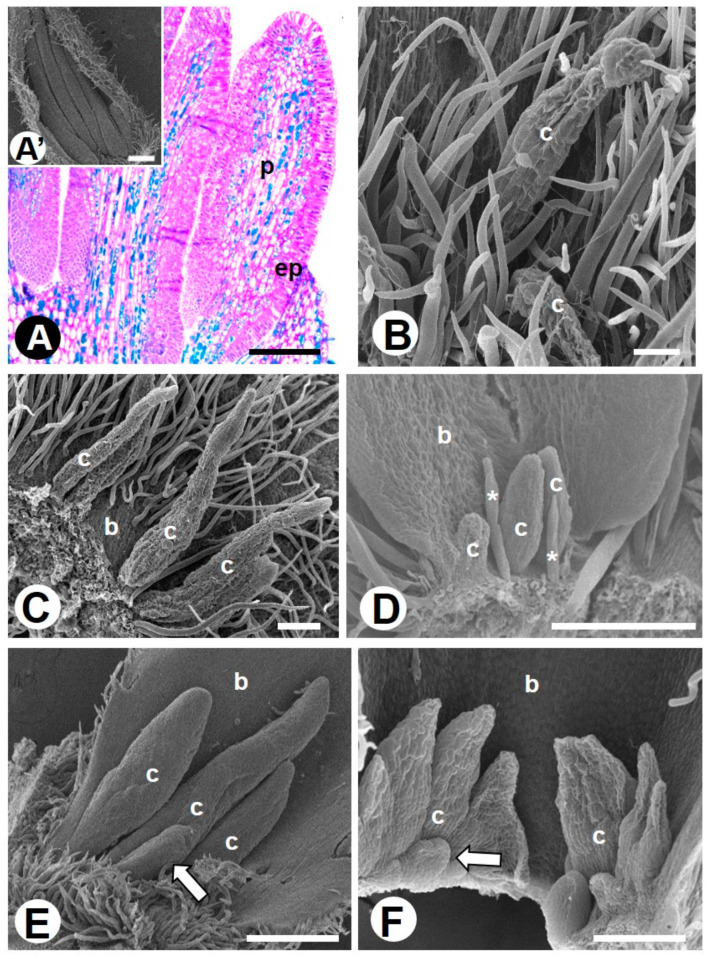
Colleters found in flowers of Rhamnaceae species. (**A’**,**B**–**F**) = SEM, (**A**) = light microscopy (LM). (**A**) Surface view (**A’**) and anatomy (**A**) of colleters in *Colubrina glandulosa*. Note the palisade-like epidermis and central parenchymatic axis containing phenolic idioblasts (greenish staining). Colleters are massive and fill the interior of the bracts. (**B**) Colleters of *Gouania latifolia*. (**C**) Colleters of *Gouania virgata* among the trichomes in the base of the bracts. (**D**) Wide colleters of *Hovenia dulcis* in the base of bracts. (**E**) Wide and small (arrow) colleters of *Rhamnidium elaeocarpum* similar to those found in *C. glandulosa*. (**F**) Wide and small (arrow) colleters of *Sarcomphalus joazeiro*. Symbols: b = bract, c = colleter, ep = epidermis, p = parenchymatic axis, * = trichomes, arrows = small colleter. Scale bars: (**A**,**D**), 200 μm; (**A’**,**E**), 500 μm; (**B**), 50 μm; (**C**,**F**), 100 μm.

**Figure 2:**
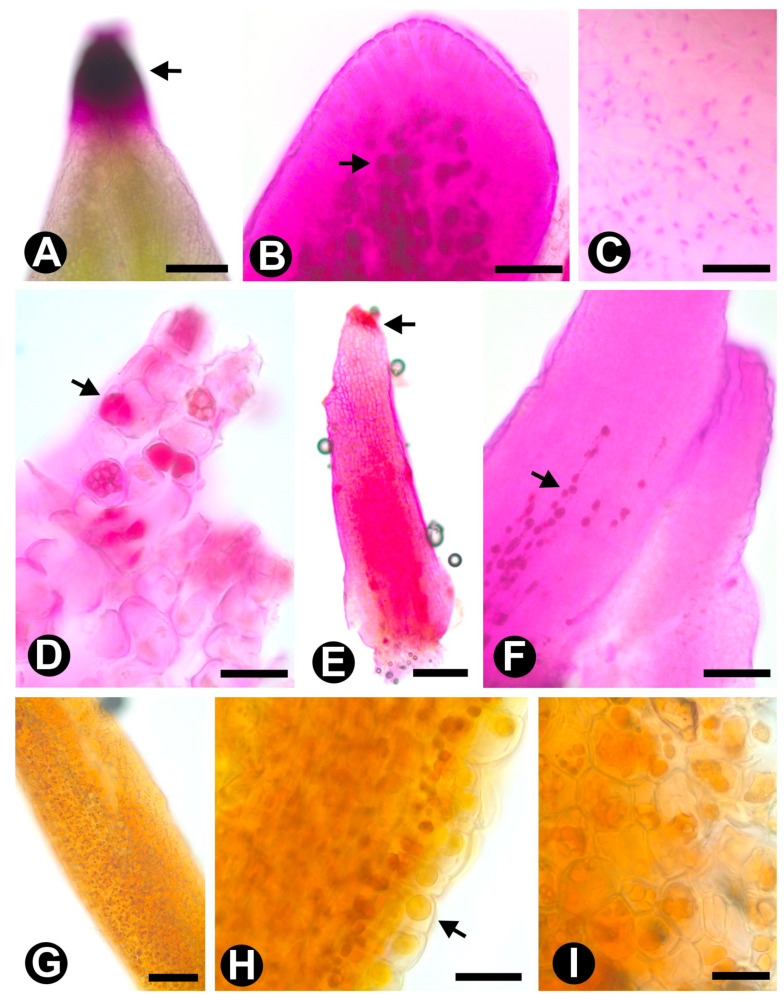
Histochemical tests applied to fresh (**A**,**E**) and fixed colleters (others) of Rhamnaceae species. (**A**–**F**) = Periodic acid and Schiff, (**G**–**I**) = Sudan III. (**A**–**C**) Colleters of *Colubrina glandulosa*; polysaccharides stained (arrows) at the top of fresh material and in the column parenchyma of fixed material. (**D**) Colleter of *Gouania latifolia* showing polysaccharides (arrow). (**E**–**F**) Colleters of *Rhamnidium elaeocarpum* with polysaccharides (arrows) distributed similarly to those found in *C. glandulosa*. (**G**–**H**) Colleteres of *Gouania virgata* showing the lipids distributed throughout the colleter and detail of oil drops (arrow) in the parenchymatic and epidermal cells. (**I**) Oil drops in the parenchyma of a *Hovenia dulcis* colleter. Scale bars: (**A**,**E**), 100 μm; (**B**,**F**,**G**), 50 μm; (**C**,**D**,**H**), 20 μm.

Colleters are emergences found on the adaxial side of floral bracts (*Colubrina glandulosa*, *Hovenia dulcis*, and *Rhamnidium elaeocarpum*) and inflorescence bracts (*Gouania latifolia*, *Gouania virgata,* and *Sarcomphalus joazeiro*). They can be found early during floral development, but they disappear during floral anthesis when the bracts senesce and then lapse. Colleters vary subtly in shape and size, although they have the same internal structure (Figure 1A–F). They are formed by a palisade-like epidermis covering a parenchymatic central axis without vascular elements (Figure 1A). The epidermal and parenchymatic cells secrete phenolic compounds (Figure 1A), mucilage (Figure 1A and Figure 2A–F), and lipids (Figure 2G–I).

**Figure 3:**
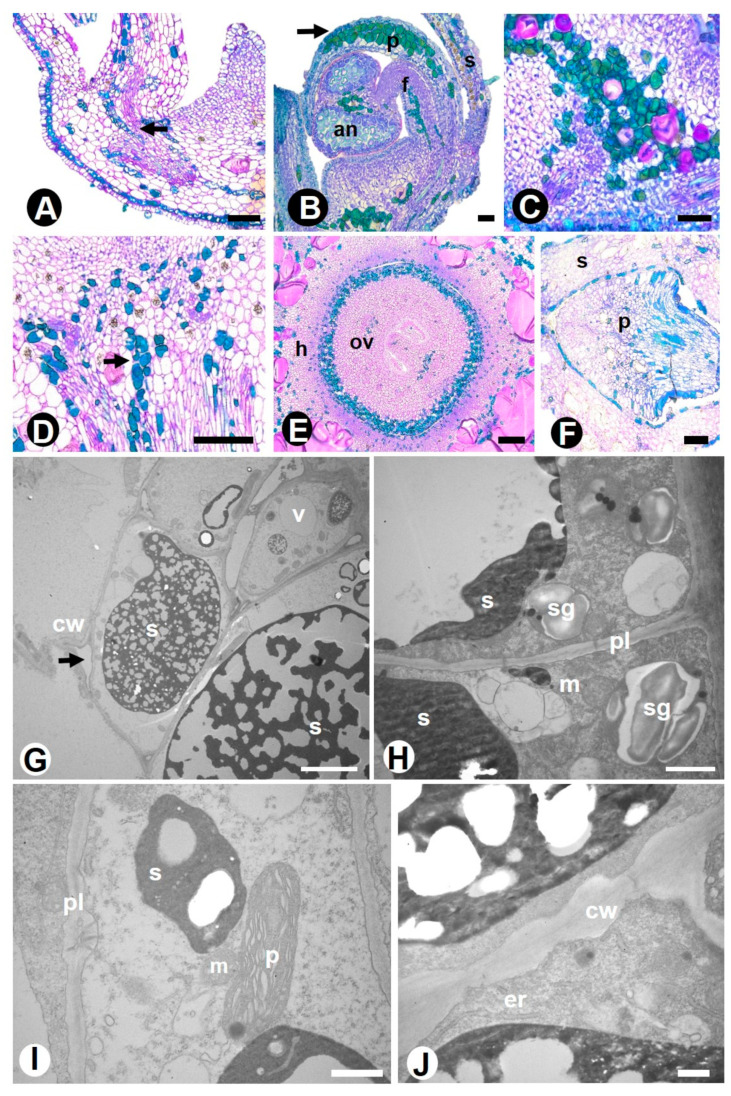
Phenolic idioblasts in the flower of Rhamnaceae species. (**A**–**F**) = LM, (**G**–**J**) = TEM. (**A**–**D**) = longitudinal sections, (**E**,**F**) = transverse sections. (**A**) *Colubrina glandulosa*—phenolic idioblasts (arrow) in the organs of the perianth, forming a subepidermal row in the hypanthium base. (**B**) *Gouania virgata*—phenolic idioblasts (arrow) forming clusters in the petal mesophyll, anther connective, and hypanthium parenchyma. (**C**) *Gouania latifolia*—phenolic idioblasts in the hypanthium parenchyma (floral cup base). (**D**) *Hovenia dulcis*—clustered phenolic idioblasts (arrow) in the hypanthium parenchyma, below the carpel and nectary. (**E**) *Rhamnidium elaeocarpum*—phenolic idioblasts surrounding the base of the ovary; note the idioblasts scattered in the hypanthium. (**F**) *Sarcomphalus joazeiro*—phenolic idioblasts in the sepal and petal parenchyma. (**G**–**H**) *H. dulcis*—phenolic idioblast with secretion in the central vacuole and several plastids with starch grains in the parietal cytoplasm. (**I**–**J**) *R. elaeocarpum*—cytoplasm with plastids, mitochondria, and vacuole with electron-dense secretion. Symbols: an = anther, cw = cell wall, er = endoplasmic reticulum, f = filament, h = hypanthium, m = mitochondria, ov = ovary, p = petal (LM), p = plastid (TEM), pl = plasmodesma, s = sepal (LM), s = secretion (TEM), sg = starch grain, v = vacuole. Scale bars: (**A**,**B**), 50 μm; (**C**–**F**), 100 μm; (**G**), 10 μm; (**H**–**J**), 2 μm.

Phenolic idioblasts (Figure 3) were widely found in all floral tissues of the six species studied. *Rhamnidium elaeocarpum* specifically has phenolic idioblasts spread throughout all floral organs. TEM analyses revealed that the phenolic compounds are produced in the cytosol (Figure 3G,H) with the involvement of plastids (Figure 3I) and then transported to the vacuole for storage (Figure 3H,J). The various mitochondria and starch grains found in these idioblasts in the pre-anthesis phase (Figure 3H,I) are involved in the energy supply for the formation and accumulation of these compounds.

**Figure 4:**
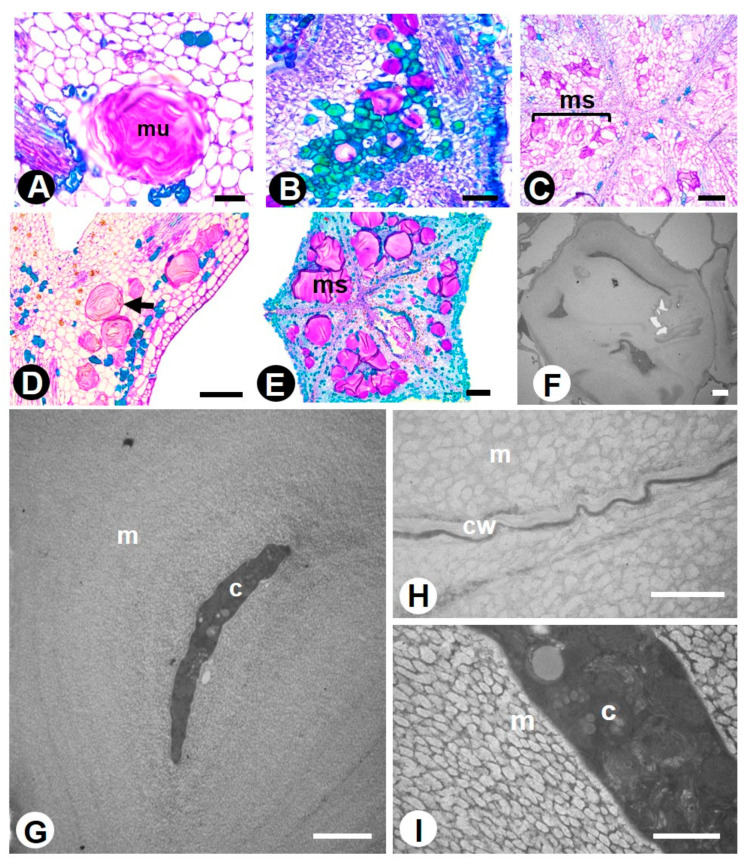
Mucilaginous idioblasts in the flower of Rhamnaceae species. (**A**–**E**) = LM, (**F**–**I**) = TEM. (**A**,**B**,**D**) = longitudinal sections, (**C**,**E**) = transverse sections. (**A**) *Colubrina glandulosa*—mucilaginous idioblast in the hypanthium parenchyma. (**B**) *Gouania virgata*—a cluster of mucilaginous idioblasts in the sepal mesophyll, as well as phenolic idioblasts in green. (**C**) *Sarcomphalus joazeiro*—a cluster of mucilaginous idioblasts forming a mucilage system. (**D**–**F**) *Hovenia dulcis*—mucilaginous idioblasts in the hypanthium parenchyma (arrow). (**F**) Dissolution of cell walls of adjacent cells and mucilage accumulation causing the cytoplasm restricted to the cell center. (**G**–**I**) *Rhamnidium elaeocarpum*—detail of a mucilage idioblast in the sepal mesophyll. Note that the same process of mucilage storage (dissolution of the cell wall and central cytoplasm) occurs. Symbols: c = cytoplasm, cw = cell wall, m = mucilage, ms = mucilage system. Scale bars: (**A**,**B**), 50 μm; (**C**–**E**), 100 μm; (**F**), 10 μm; (**G**,**H**), 2 μm; I, 3 μm.

Mucilaginous idioblasts are also ubiquitous in the sampled species (Figure 4) and are widely distributed in floral tissues. In *Colubrina glandulosa,* they form a row of subepidermal cells in the hypanthium basis and are also found in the subepidermis and mesophyll of perianth organs. *Gouania virgata* has clusters mainly in the sepal mesophyll, in the anther connective, and in the parenchyma below the nectary. *Hovenia dulcis* has idioblasts in the hypanthium parenchyma, clustered in the region of the floral cup base. In *Sarcomphalus joazeiro,* they are clustered in the hypanthium parenchyma, just below the gynoecium and nectary. In *Rhamnidium elaeocarpum,* they are found in all floral organs but densely clustered in the parenchyma of sepals, the anther connective, and in the subepidermal layers of the carpel. TEM analysis showed that the mucilage accumulates between the cell wall and the plasma membrane, gradually compressing the protoplast to the center of the cell (Figure 4F–I). The presence of several active Golgi bodies in the cytoplasm indicates its evolvement in mucilage production (Figure 4I).

All types of idioblasts (phenolic, mucilaginous) have unlignified cell walls and a dense protoplast.

**Figure 5:**
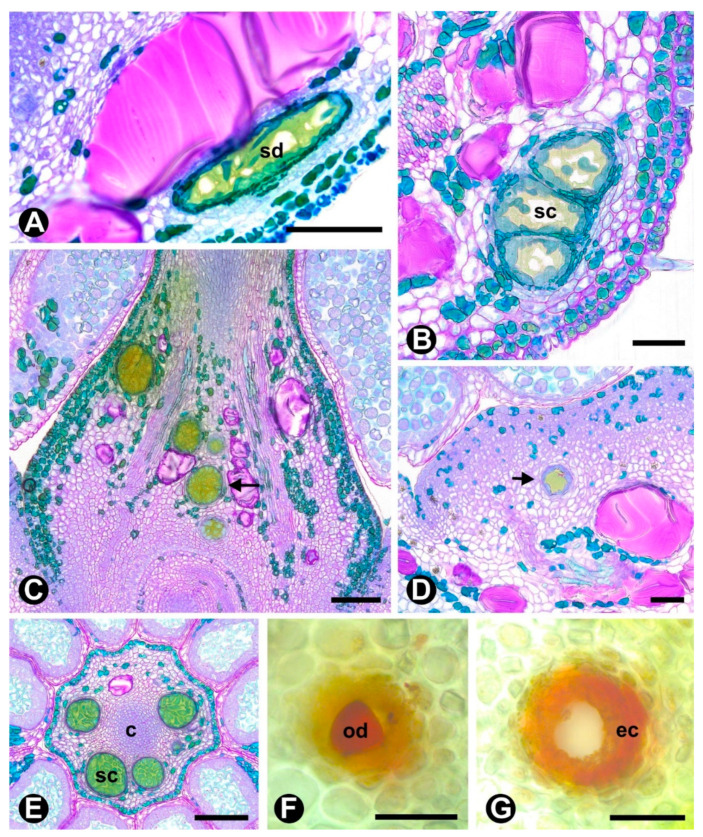
Secretory cavities and ducts in the flower of *Rhamnidium elaeocarpum*. (**A**,**C**,**D**) are longitudinal sections; (**B**,**E**) are transverse sections; and (**F**–**G**) free-hand sections. (**A**–**E**) = Toluidine Blue, (**F**–**G**) = Sudan III. (**A**–**E**) Secretory cavities and ducts (arrows) in the hypanthium (**A**,**B**), ovary (**C**,**E**), and below the nectariferous parenchyma (**D**). (**F**–**G**) Positive result for Sudan III revealing the lipids inside the lumen and secretory epithelial cells. Symbols: c = gynoecial compitum, ec = epidermal cells, od = oil drop, sc = secretory cavity, sd = secretory duct. Scale bars: (**A**,**C**,**E**), 100 μm; (**B**,**D**,**F**,**G**), 50 μm.

Oil-resin cavities and ducts were exclusively found in *Rhamnidium elaeocarpum*. The cavities were mostly observed in the ovarian parenchyma, above the ovules, and were sparsely present in the hypanthium base. These structures consist of an isodiametric lumen lined with secretory epithelial cells (Figure 5). Secretory ducts occur only in the pedicel and hypanthium base. The ducts have an elongated lumen delimited by secretory epithelial cells.

### 2.2. Data Obtained from the Literature

We found data on secretory structures (excluding nectaries) for 257 taxa of the Rhamnaceae family, covering almost all tribes (see Table S1). The bibliographic survey carried out revealed that the family has a wide range of cells that store mucilage: epidermis, hypodermis, idioblasts. Most species show a mucilage-secreting epidermis in the leaves. Mucilage reservoirs, which are interpreted as cavities or ducts by many authors, are also common in the leaf veins but were not found in the species of the tribes Pomaderreae, Colletieae and Phyliceae, or the genera *Ceanothus* (Inserteae sedis), *Crumenaria*, *Helinus*, *Reissekia* (Gouanieae), *Berchemia*, *Krugiodendron*, *Pseudoziziphus*, *Reynosia,* and *Rhamnus* (Rhamneae, except *Rhamnus diffusa*). In addition, most species show tannin-secreting cells, which can occur in the mesophyll, epidermis, and especially in the parenchymal sheath of vascular bundles. Most species show mucilage and tannins in their tissues, which normally accumulate in different cells. However, all genera of the Colletieae tribe and *Berchemia* (Rhamneae) display idioblasts that accumulate these two types of compounds. There are also some genera in the family that do not contain mucilage but only contain tannin idioblasts, such as *Helinus* (Gouanieae), *Noltea*, *Phylica* (Phyliceae), *Krugiodendron,* and *Rhamnus* (Rhamneae). Other types of secretory structures such as resin ducts occasionally occur in some related genera of the Rhamneae tribe (*Auerodendron*, *Karwinskia*, *Reynosia,* and *Rhamnidium*). Additionally, there are reports of glandular leaf teeth for some genera such as *Ceanothus*, *Phylica*, *Noltea*, *Rhamnus,* and *Sageretia*, although they are unexplored.

## 3. Discussion

Rhamnaceae is a poorly studied group in terms of morphology and functionality of floral secretory structures. The present study updated the occurrence of these structures in the family and contributed to the knowledge of their morphology and systematic significance. Furthermore, the information reported here about the structure, secreted content, and occurrence of the secretory structures of Rhamnaceae helped us to understand the assertive folk use of its species. Studies on pollination biology integrated with studies of development and morphology will certainly provide answers about the evolution and the role of these structures in the reproductive process of the group.

### 3.1. An Overview of the Secretory Structures of Rhamnaceae

**Figure 6:**
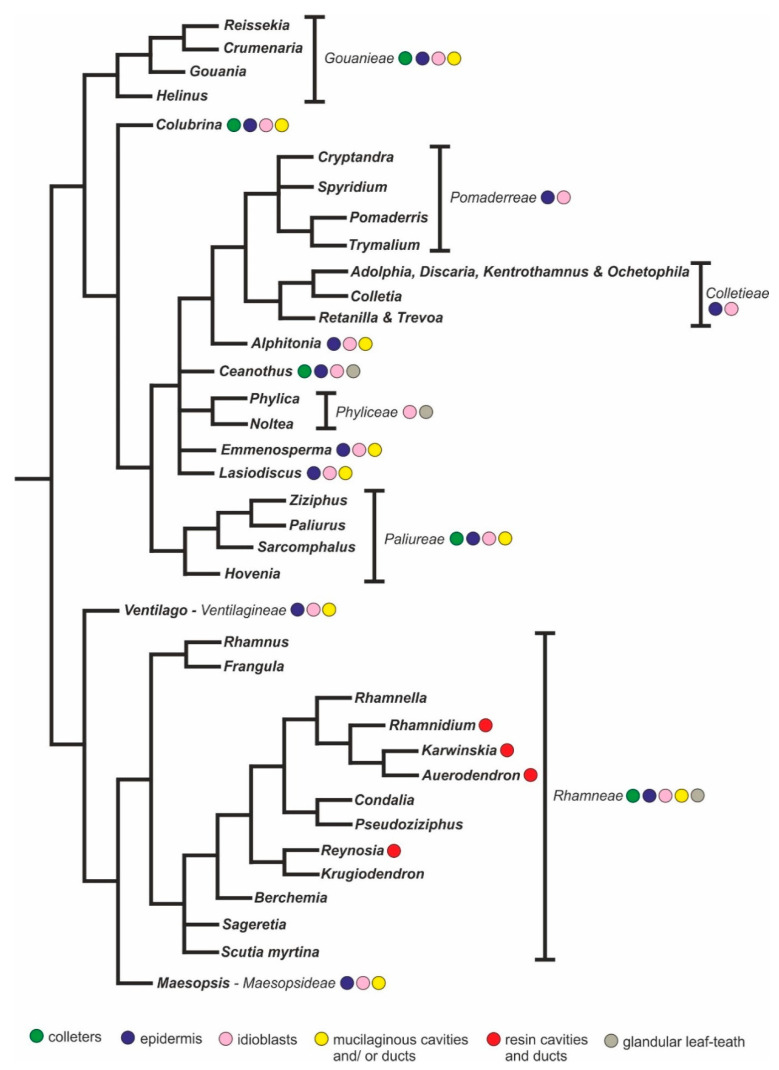
Cladogram based in Hauenschild et al. [45] showing the phylogenetic relations of Rhamnaceae representatives. The occurrence of secretory structures in the Rhamnaceae species is marked in the topography. Data extracted from the literature (see Table S1) and from the present study.

Over the last 30 years, research on the floral secretory structures of Rhamnaceae has been based primarily on the nectaries due to this remarkable and even diagnostic morphological trait. In addition, there is a great deal of knowledge about the morphology and distribution of mucilage and tannin-secreting structures in the vegetative organs of several species of the family [10,11,12]. These studies have revealed that the type and location of the leaf secretory structures have a strong taxonomic significance at the genus or tribe levels. However, these studies were published over one hundred years ago, which impels us to reevaluate the systematic significance of these structures in the light of the most recent phylogeny published for the family (Figure 6).

We found several types of internal and external secretory structures widely distributed among the floral organs of the sampled species. The external secretory structure found in Rhamnaceae consists of multicellular emergences located on the adaxial surface of involucral bracteoles of floral meristems and bracts of inflorescences. Considering the concept found in the literature [38,46], we named such structures colleters. The main functions attributed to colleters are to ensure lubrication of developing organs and to promote protection against dehydration, pathogens, and herbivores [46]. To confirm these functions, it is important to elucidate the chemical composition of the secreted content of colleters. This is because, traditionally, colleters have been designated as glands present on the surface or axil of vegetative and/or reproductive developing organs that secrete a sticky substance primarily containing mucilage or lipophilic compounds, or both [38,46]. Our histochemical tests account for the presence of mucilage and lipids, as well as the presence of phenolic compounds, regardless of the species, morphology, or position of the colleters. So, we document for the first time colleters in floral organs of Rhamnaceae, their anatomy, and the type of secreted compounds. Colleters are structures poorly recorded in the Rosales order (they were previously found in Moraceae) [47]. Thus, it is difficult to establish possible structural homologies for the order.

Four types of internal secretory structures were recognized in the present study according to their chemical composition: phenolic idioblasts, mucilaginous idioblasts, and oil-resin cavities and ducts.

Secretory idioblasts are isolated cells that may vary in shape, size, and content. Idioblast is a general convenient term used to designate an array of cell types because of the impossibility of providing a specific morphological or physiological description for them [48]. Thus, secretory idioblasts are remarkable cells that may have different contents. The phenolic compounds found in the idioblasts are molecules traditionally related to plant defense. Considering the variation in phenolic structure and concentration, these compounds may cause feeding deterrence or stimulation, digestion inhibition or stimulation, toxicity, disease resistance, signal inhibition, signal transduction, and nutrient cycle regulation. Furthermore, phenols can be used as potential biomarkers of pollution because they participate in the plant response to heavy metal accumulation, acting as antioxidants able to cleanse free radicals produced by metal ions [49,50,51,52]. The function of mucilage may consist of carbohydrate storage, water storage, reduction of transpiration, protection against intensive radiation by light scattering or reflection, and protection against herbivory [53]. Mucilaginous idioblasts are notable cells in the floral tissues of Rhamnaceae species because they are voluminous and widely distributed. As floral organs, they were previously registered only for *Reynosia* species (Table S1), so that the present record shows that floral mucilage idioblasts, as well as leaf mucilage idioblasts, probably have a wider distribution in Rhamnaceae. We interpret that these remarkable cells are actually formed by several cells whose walls were dissolved, forming a large mucilaginous system. The ontogeny of a mucilage system and storage of the contents was detected with the aid of TEM. The process can be traced as follows: (1) the idioblasts emerge as single small cells that produce mucilage and then grow in size and volume, forming rows; (2) at a certain stage, the outer part of the wall, i.e., that which is in contact with the adjacent cell wall, starts to dissolve, resulting in a very large and evident structure; (3) the proximity of these units results in a mucilage system; (4) with progressive mucilage deposition, the interior may become almost occluded with mucilage and the protoplast be confined to thread-like regions. Mucilage is scarcely found in Rosales (Moraceae, Ulmaceae, and Rosaceae) and is generally associated with colleter secretion and vegetative organs. Interesting is the presence of idioblasts that accumulate tannins and mucilage in the same type of cell in the tribe Colletieae, a characteristic that can be a synapomorphy for the tribe. The presence of phenolic idioblasts and mucilage in addition to non-glandular trichomes and flesh perianth organs suggests that these floral traits may be related to the occurrence of plants in xeric areas. The mixed nature of the composition of colleter secretion leads us to hypothesize that mucilage and lipids respectively act against desiccation and on the maintenance of the position of the developing organ. Terpenes were not found in the studied species. In Rosales, this class of compound is present in the laticifers of representatives of Moraceae (e.g., *Ficus* species) [41] and Cannabaceae (*Celtis*, *Pteroceltis,* and *Trema* species) [54].

Cavities and ducts are characterized by the presence of a large intercellular space (lumen) lined with a specialized secretory epithelium consisting of several cells. The format of the lumen characterizes a cavity (isodiametric lumen) or a duct (elongated lumen) [38,39]. A range of compounds such as oils, resins, phenolics, polysaccharides, and others may be synthesized by the epithelial cells of cavities and ducts. The presence of cavities and ducts in *Rhamnidium elaeocarpum* was confirmed in longitudinal and transverse sections. These glands were filled with a lipid granular-like secretion. The secretion is stored in the lumen and does not come into contact with any surrounding tissue. Its release onto the environment occurs only by rupture of the secretory system. We verified that these floral cavities and ducts are filled with oil, as also observed in *R. elaeocarpum* leaves and seeds [11,37]. Resin cavities and ducts have been described for the leaves of the phylogenetically related species *Auerodendron reticulatum*, *Karwinskia*, *Reynosia revoluta,* and *Rhamnidium glabrum* (Table S1, Figure 6). For Rhamnaceae, cavities and ducts of mucilage have also been recorded for several species. We believe that these structures are actually groups of idioblasts that have undergone lysis and form a large reservoir of mucilage. The resorption of the idioblast walls constitutes one of the most typical aspects of the formation of lysogenic pockets and has already been described for some Rhamnaceae [55,56]. The presence of flattened cells surrounding the aged pouches is a common point with oil-resin cavities and ducts, but in the first case, the bordering cells are devoid of secretory activity. Even so, these structures were interpreted by Fahn [38] as mucilage cavities or ducts. Furthermore, these structures in their final form cannot be called idioblasts since, with the dissolution of the cell walls, the mucilage is finally stored in an extracellular medium. Thus, checking the morphology of these glands is important due to their potential systematic significance. For example, Table S1 and Figure 6 show that mucilaginous cavities and ducts seem to be absent in the phylogenetically related taxa *Ceanothus*, Colletieae, Phyliceae, and Pomaderreae; which can be a common feature for this group. In addition, the presence of mucilaginous cells, cavities, and ducts in species of the genus *Frangula* may be used as a diagnostic condition to support the recent separation of these species of the genus *Rhamnus* [57], for which only tannin idioblasts have been reported.

Previous reports of glandular leaf teeth were found for *Ceanothus* sp. (*Incertae sedis*), *Noltea africana*, *Phylica nitida* (Phyliceae), *Rhamnus* sp., and *Sageretia thea* (Rhamneae) (Table S1, Figure 6. According to the phylogeny proposed by Hauenschild and collaborators [45], the Phyliceae tribe and *Ceanothus* seem to be closely related. However, the morphology of these structures seems to be diverse, so that, while in *Ceanothus,* they resemble secretory trichomes [58], in *Noltea africana* and *Rhamnus alpinus,* they consist of a set of glandular cells from the epidermis and parenchyma [12]. However, the glandular hairs in *Ceanothus* probably are emergences since vascular bundles are reported in the stalk of such secretory structure [12]. New morphological and ontogenetic studies of the glandular leaf teeth of Rhamnaceae are welcome to elucidate the diversity of cell types that these glands present, as well as the type and process of secretion, which are currently unknown. Such knowledge could bring new information about the ecology and systematics of the group.

In a general review, Rhamnaceae flowers may produce nectar, phenols, polysaccharides (including mucilage), and oils. It is impressive that this inconspicuous flower can contain such a wide range of secretory structures as well as a chemical variety of secretions.

In Rhamnaceae, a monophyletic family supported by combining *trn*L-*trn*F and nuclear ribosomal internal transcribed spacer (ITS) sequence data [45,59], secretory structures are found in different lineages (Figure 6). Groups within Rhamnaceae exhibit a differentiated set of floral characters ranging from ovary characteristics to fruit type, which have been used in infrafamilial circumscription. In contrast, the presence of mucilaginous epidermis and idioblasts appears to be widespread in the family, in contrast to other families of the Order Rosales (Moraceae, Ulmaceae, and Rosaceae), which lack this type of secretory structure. In addition, our results show that the presence of a particular type of secretory structure, or group of secondary compounds, coincide with the delimitation of some genera, tribe, or phylogenetically related group of Rhamnaceae, which were previously determined by molecular biology studies, and therefore, these characteristics can also be used in its intrafamily circumscription.

Secretory structures are probably responses acquired during the diversification of lineages to satisfy some special needs related to biotic or abiotic environmental stimuli. Plants usually use a broad chemical arsenal for defense against environmental factors [60,61,62]. They synthesize different compounds, sometimes in specific forms for defense, which might include aspects of nutritional quality (e.g., proteins and antiproteins), phenology, and tolerance that may provide direct (morphological characteristics, e.g., spines, trichomes, and leaf toughness) or indirect (e.g., volatiles and branching architecture) types of defense. If a trait has independently evolved multiple times, it is likely that the selective pressures driving the evolution of these convergent adaptations are common and widespread. However, if closely related species share a trait due to common ancestry, then an adaptive advantage does not necessarily need to be involved. As Rhamnaceae is a cosmopolitan family whose species inhabit different environments, and the same type of secretory structure can perform different ecological functions, it is difficult to make associations between the appearance of these structures in a group with the ecological niche of its species.

### 3.2. Outlook

The knowledge of the secretory structures of plants involves the investigation of specific parameters such as structure, chemical nature of the exudate, and function. In this respect, our study focuses on the information obtained from the floral gland structure and chemical nature of the exudate of Rhamnaceae representatives, except for the well-known floral nectary. We also linked data from different sources about this subject to family representatives, extrapolating the considerations to species not studied here and even from other taxonomic groups.

## 4. Materials and Methods

### 4.1. Plant Material

Novel data were obtained from *Colubrina glandulosa* subsp. *reitzii* (M. C. Johnst) Bhoridi, *Gouania latifolia* Reissek, *Gouania virgata* Reissek, *Hovenia dulcis* Thunb., *Rhamnidium elaeocarpum* Reissek, and *Sarcomphalus joazeiro* (Mart.) Hauenschild. Samples were collected and georeferenced and voucher specimens were deposited in the SPFR herbarium (Universidade de São Paulo, Ribeirão Preto Campus, Brazil) and the RB herbarium of the Jardim Botânico do Rio de Janeiro (Rio de Janeiro, Brazil) (Table 2).

The valid scientific names of species were confirmed in Plants of the World online [63]. The clade and tribe classifications adopted follow Hauenschild et al. [45].

### 4.2. Preparation and Fixation of Samples

Floral buds in different stages before anthesis and flowers were fixed in buffered formaldehyde [64] for 24 h, stored in 70% alcohol, and prepared for surface, anatomical, and histochemical analysis.

### 4.3. Surface Analysis (SEM)

The surface of external secretory structures was analyzed by scanning electron microscopy (SEM). The samples were dehydrated in an ethanol series, critical-point dried in a Bal-Tec CPD 030 (Balzers, Oberland, Liechtenstein), mounted on metal stubs, adhered to carbon adhesive tape, and then covered with gold in a Bal-Tec SCD 050 sputter-coater. Observations and illustrations were made using a Zeiss EVO-50 scanning electron microscope at 15 kV.

### 4.4. Anatomical Analysis (LM)

For the anatomical exam (light microscopy—LM), samples were dehydrated in an ethanol series, embedded in histological resin [65], and transversely and longitudinally sectioned with 3 or 3.5 μm thick in a RM 2245 rotary microtome (Leica, Wetzlar, Germany). The sections were stained with toluidine blue in phosphate buffer, pH 5.8 [66], and mounted. Observations and illustrations were obtained using a Leica DM 4500 B light microscope connected to a Leica DFC 320 digital camera. Scales were determined under the same optical conditions.

Samples embedded in historesin were stained with toluidine blue for the detection of phenolic compounds and mucilage [66]. Alcohol-stored and fresh material was free-hand sectioned and stained with period acid and Schiff (PAS) for neutral polysaccharides [67], with Sudan III and Sudan IV [68] for total lipids, and with Nadi reagent [69] for essential oils and oil-resin localization.

### 4.5. Ultrastructural Analysis (TEM)

Floral organs of *Hovenia dulcis* and *Rhamnidium elaeocarpum* were chosen for ultrastructural analysis of internal secretory structures (transmission electron microscopy—TEM). For this purpose, small sections of hypanthium were fixed in Karnovsky’s solution [70] for 24 h, post-fixed in 1% osmium tetroxide in 0.1 M phosphate buffer, pH 7.2, washed in distilled water, dehydrated, and embedded in Araldite epoxy resin. Sections were obtained with a Reichert Ultracut S ultramicrotome (Leica, Wien, Austria) at 60–70 nm, collected on copper grids, and contrasted with 2% uranyl acetate and lead citrate for 15 min. Transmission electron micrographs were obtained using a Jeol 100CXII instrument.

## Figures and Tables

**Table 1:** Occurrence of secretory structures in the flowers of the studied species of Rhamnaceae. Symbols: (-) = absence; (+) = presence.

Species	Colleter	Cavity	Duct	Idioblast
*C. glandulosa*	+ (bract)	-	-	+ (bract, pedicel, hypanthium, sepal, petal, filament, connective, anther, carpel)
*G. virgata*	+ (bract)	-	-	+ (bract, pedicel, hypanthium, sepal, petal, filament, connective, anther, carpel)
*G. latifolia*	+ (bract)	-	-	+ (bract, pedicel, hypanthium, sepal, petal, filament, connective, anther, carpel)
*H. dulcis*	+ (bract)	-	-	+ (bract, pedicel, hypanthium, sepal, petal, filament, connective, anther, carpel)
*R. elaeocarpum*	+ (bract)	+ (hypanthium, carpel)	+ (pedicel, hypanthium)	+ (bract, pedicel, hypanthium, sepal, petal, filament, connective, anther, carpel)
*S. joazeiro*	+ (bract)	-	-	+ (bract, pedicel, hypanthium, sepal, petal, filament, connective, anther, carpel)

**Table 2:** Rhamnaceae species sampled in the present study.

Species	Clade	Tribe	Vouchers	Collection Sites
*Colubrina glandulosa*	Ziziphoid	*Incertae sedis*	SPFR 17155	Ribeirão Preto, SP, Brazil (FORP ^1^-USP ^2^).
*Gouania virgata*	Ziziphoid	Gouanieae	SPFR 17156	Ribeirão Preto, SP, Brazil (Santa Tereza Forest).
*Gouania latifolia*	Ziziphoid	Gouanieae	Cortez, 70	Brasília-DF, Brazil (National Park of Brasília).
*Hovenia dulcis*	Ziziphoid	Paliureae	SPFR 17157	Ribeirão Preto, SP, Brazil (CREU ^3^-USP).
*Rhamnidium elaeocarpum*	Rhamnoid	Rhamneae	SPFR 17158	Ribeirão Preto, SP, Brazil (CEFER ^4^-USP).
*Sarcomphalus joazeiro*	Ziziphoid	Paliureae	RB 6544	Rio de Janeiro, RJ, Brazil (JBRJ ^5^).

^1^ Faculdade de Odontologia de Ribeirão Preto; ^2^ Universidade de São Paulo. ^3^ Conjunto Residencial dos Estudantes Universitários; ^4^ Centro de Esportes e Educação Física de Ribeirão Preto; ^5^ Jardim Botânico do Rio de Janeiro.

## Data Availability

Data sharing is not applicable to this article.

## Supplementary Materials

The following data are available online at https://www.mdpi.com/article/10.3390/plants10040736/s1, Table S1: List of secretory structures found in Rhamnaceae (excluding the nectaries), showing the compounds and organ of occurrence. Data extracted from the literature and from the present study (species in bold). * The mucilaginous ducts, when present in the leaves, always occur in the collenchyma of the veins, except in *Maesopsis*, where they also occur in the phloem.
