# Supplementary material for: Uncovering the Neglected Floral Secretory Structures of Rhamnaceae and Their Functional and Systematic Significance
Source: Plants (Basel). 2021 Apr 9;10(4):736. doi: 10.3390/plants10040736 (PMC8070232; doi:10.3390/plants10040736)
Supplement: Supplementary file 1 [file plants-10-00736-s001.pdf]

Table S1. List of secretory structures found in Rhamanaceae (excluding the nectaries), showing the compounds and organ of occurrence. Data extracted from the literature and from the present study (species in bold). * The mucilaginous ducts, when present in the leaves, always occur in the collenchyma of the veins, except in *Maesopsis*, where they also occur in the phloem.

Tribe	Species	Secretory structure	Compounds	Organ	References
Incerteae sedis	*Alphitonia sp.*	Epidermis, idioblasts, cavities, ducts	Mucilage	Leaf (blade, petiole)	12, 13
	Alphitonia excelsa	Epidermis, ducts, osmophores	Mucilage, terpenes	Flower, leaf (blade)	10, 24
	Ceanothus sp.	Glandular leaf-teeth, Epidermis, hypodermis, idioblasts, colleters	Mucilage, tannins	Flower, leaf (blade, petiole)	12, 13, 46, 73
	Ceanothus americanus	Idioblasts	Mucilage	Leaf (blade, petiole), stem	74
	Ceanothus buxifolius	Epidermis, idioblasts	Mucilage, tannins	Leaf (blade)	10
	Ceanothus caeruleus	Idioblasts	Tannins	Leaf (blade)	10
	Ceanothus cordulatus	Epidermis, idioblasts	Mucilage, tannins	Leaf (blade)	10
	Ceanothus crassifolius	Epidermis; hypodermis	Mucilage, tannins	Leaf (blade)	10, 12
	Ceanothus cuneatus	Epidermis	Mucilage	Leaf (blade)	10
	Ceanothus dentatus	Glandular leaf-teeth (trichomes)	Lipids, flavonoids	Leaf (blade)	60
	Ceanothus foliosus	Glandular leaf-teeth (trichomes)	Lipids, flavonoids	Leaf (blade)	60
	Ceanothus hearstiorum	Glandular leaf-teeth (trichomes)	Lipids, flavonoids	Leaf (blade)	60

Ceanothus herbaceus	Idioblasts	Mucilage	Leaf (blade, petiole), stem	74
Ceanothus impressus	Glandular leaf-teeth (trichomes)	Lipids, flavonoids	Leaf (blade)	60
Ceanothus integerrimus	Epidermis	Mucilage	Leaf (blade)	10
Ceanothus oliganthus	Glandular leaf-teeth (trichomes); epidermis	Mucilage, lipids, flavonoids	Leaf (blade)	10, 60
Ceanothus oliganthus subsp. sorediatus	Idioblasts	Mucilage, tannins	Leaf (blade)	10
Ceanothus papillosus	Glandular leaf-teeth (trichomes), idioblasts	Mucilage, lipids, flavonoids	Leaf (blade)	10, 12, 60
Ceanothus prostratus	Epidermis	Mucilage	Leaf (blade)	10
Ceanothus sanguineus	Idioblasts	Mucilage, tannins	Leaf (blade)	10
Ceanothus x *veitchianus*	Glandular leaf-teeth (trichomes)	Lipids, flavonoids	Leaf (blade)	60
Ceanothus velutinus	Glandular leaf-teath (trichomes)	Lipids, flavonoids	Leaf (blade)	12, 60
Colubrina sp.	Epidermis, idioblasts, cavities, ducts	Mucilage, tannins	Leaf (blade, petiole)	10, 12, 13
Colubrina arborescens	Idioblasts, ducts	Mucilage	Leaf (blade)	10
Colubrina asiatica	Idioblasts, cavities	Tannins	Leaf (petiole)	12
Colubrina glandulosa	Colleters, idioblasts	Mucilage, phenols, terpens	Flower	Present study

	Colubrina greggii	Ducts	Mucilage	Leaf (blade)	10
	Colubrina heteroneura	Epidermis, ducts	Mucilage, tannins	Leaf (blade)	10
	Colubrina obscura	Epidermis, ducts	Mucilage	Leaf (blade)	11
	Colubrina spinosa	Epidermis, ducts	Mucilage, tannins	Leaf (blade)	10
	Colubrina triflora	Ducts	Mucilage	Leaf (blade)	10
	Emmenosperma sp.	Epidermis, idioblasts, cavities	Mucilage	Leaf (blade, petiole)	13
	Emmenosperma alphitonoides	Epidermis, ducts	Mucilage	Leaf (blade)	11
	Lasiodiscus sp.	Epidermis, idioblasts, cavities, ducts	Mucilage	Leaf (blade, petiole)	12, 13
	Lasiodiscus mannii	Epidermis, ducts	Mucilage, tannins	Leaf (blade)	10
Colletieae	*Adolphia infesta*	Idioblasts	Mucilage, tannins	Leaf (blade)	75
	Colletia sp.	Epidermis, idioblasts	Mucilage	Leaf (blade)	13
	Colletia hystrix	Idioblasts	Mucilage, tannins	Leaf (blade)	75
	Colletia paradoxa	Idioblasts, osmophores	Mucilage, terpenes	Flower	24
	Discaria sp.	Epidermis, idioblasts	Mucilage, tannins	Leaf (blade)	12, 13, 15
	Discaria americana	Epidermis, idioblasts, osmophores	Mucilage, tannins, terpenes	Flower, leaf (blade)	10, 24

	Discaria chacaye	Idioblasts	Mucilage, tannins	Leaf (blade)	10, 12
	Discaria pubescens	Idioblasts	Mucilage, tannins	Leaf (blade)	10
	Discaria × serratifolia	Idioblasts	Mucilage, tannins	Leaf (blade)	10, 12
	Kentrothamnus weddellianus	Epidermis, idioblasts	Mucilage, tannins	Leaf (blade)	10, 14
	Ochetophila nana	Idioblasts	Mucilage, tannins	Leaf (blade)	10
	Ochetophila trinervis	Idioblasts	Mucilage, tannins	Leaf (blade)	10
	Retanilla ephedra	Epidermis, idioblasts	Mucilage, tannins	Leaf (blade)	16
	Retanilla patagonica	Epidermis, idioblasts	Mucilage, tannins	Leaf (blade)	16
	Retanilla stricta	Hypodermis, idioblasts	Mucilage, tannins	Leaf (blade)	16
	Retanilla trinervia	Epidermis	Mucilage	Leaf (blade)	10, 16
	Trevoa sp.	Epidermis, idioblasts	Mucilage, tannins	Leaf (blade)	12, 13, 15
	Trevoa quinquenervia	Epidermis, idioblasts	Mucilage, tannins	Leaf (blade)	10, 76
Gouanieae	*Crumenaria sp.*	Epidermis, idioblasts	Mucilage	Leaf (blade)	12, 13
	Crumenaria decumbens	Epidermis, idioblasts	Mucilage, tannins	Leaf (blade)	10
	Crumenaria erecta	Epidermis, idioblasts	Mucilage, tannins	Leaf (blade)	10
	Gouania sp.	Epidermis, idioblasts, cavities, ducts	Mucilage	Leaf (blade, petiole)	12, 13

Gouania blanchetiana	Ducts	Mucilage	Leaf (blade)	10
Gouania cornifolia	Epidermis	Mucilage	Leaf (blade)	10
Gouania corylifolia	Idioblasts	Mucilage	Leaf (blade)	10
Gouania discolor	Epidermis	Mucilage	Leaf (blade)	10
Gouania inornata	Epidermis	Mucilage	Leaf (blade)	10
Gouania latifolia ***G. virgata***	Colleters, idioblasts	Mucilage, phenols	Flower	Present study
Gouania leptostachya	Ducts	Mucilage	Leaf (blade)	10
Gouania longipetala	Epidermis, ducts	Mucilage	Leaf (blade)	10
Gouania lupuloides	Epidermis, ducts	Mucilage	Leaf (blade)	10
Gouania microcarpa	Epidermis, idioblasts, cavities, ducts	Mucilage, tannins	Leaf (blade, petiole)	10, 20
Gouania napalensis	Ducts	Mucilage	Leaf (blade)	10
Gouania nematostachya	Epidermis, ducts	Mucilage	Leaf (blade)	10
Gouania polygama	Idioblasts, ducts	Mucilage	Leaf (blade)	10
Gouania pyrifolia	Ducts	Mucilage	Leaf (blade)	10
Gouania riparia	Epidermis, ducts	Mucilage	Leaf (blade)	10
Gouania tiliifolia	Epidermis, ducts	Mucilage	Leaf (blade)	10

	Gouania tiliifolia subsp. glandulosa	Ducts	Mucilage	Leaf (blade)	10
	Gouania urticifolia	Ducts	Mucilage	Leaf (blade)	10
	Helinus brevipes	Idioblasts	Tannins	Leaf (blade)	10
	Helinus integrifolius	Idioblasts	Tannins	Leaf (blade)	10
	Helinus lanceolatus	Idioblasts	Tannins	Leaf (blade)	10
	Helinus mystacinus	Idioblasts	Tannins	Leaf (blade)	10
	Helinus spartioides	Idioblasts	Tannins	Leaf (blade)	10
	Reissekia sp.	Epidermis, idioblasts	Mucilage	Leaf (blade)	12, 13
	Reissekia cordifolia	Idioblasts	Tannins	Leaf (blade)	10
Maesopsideae	*Maesopsis sp.*	Epidermis, idioblasts, cavities, ducts	Mucilage, tannins	Leaf (blade, petiole)	12, 13
	Maesopsis eminii	Epidermis, idioblasts, ducts	Mucilage, tannins	Leaf (blade, phloem*)	11, 12
Paliureae	*Hovenia sp.*	Epidermis, idioblasts, cavities, ducts	Mucilage	Leaf (blade, petiole)	12, 13
	Hovenia dulcis	Colleters, idioblasts, ducts, food bodies, osmophores	Mucilage, phenols, tannins, lipids, terpenes	Flower, leaf (blade)	Present study, 10, 24, 77
	Paliurus sp.	Epidermis, idioblasts, cavities, ducts	Mucilage	Flower, leaf (blade, petiole)	12, 13, 73

Paliurus ramosissimus	Epidermis, idioblasts, ducts	Mucilage, tannins	Leaf (blade)	11
Paliurus spina-christi	Epidermis, idioblasts, ducts, osmophores	Mucilage, tannins, terpenes	Flower, leaf (blade, petiole), stem	11, 12, 24, 78
Sarcomphalus crenatus	Idioblasts	Tannins	Leaf (blade)	11
Sarcomphalus domingensis	Idioblasts	Tannins	Leaf (blade)	11
Sarcomphalus joazeiro	Colleters, idioblasts	Mucilage, phenols, tannins, saponins, steroids, triterpenes	Flower, stem (bark)	Present study, 79
Sarcomphalus laurinus	Idioblasts	Tannins	Leaf (blade)	11
Sarcomphalus mistol	Idioblasts, cavities, ducts	Mucilage, tannins, saponins	Leaf (blade, petiole)	11, 80
Sarcomphalus obtusifolius	Epidermis, idioblasts	Mucilage, tannins	Leaf (blade)	11
Sarcomphalus platyphyllus	Idioblasts	Tannins	Leaf (blade)	11
Sarcomphalus reticulatus	Idioblasts	Tannins	Leaf (blade)	11
Ziziphus sp.	Epidermis, idioblasts, cavities, ducts	Mucilage, tannins	Leaf (blade, petiole)	12, 13
Ziziphus abyssinica	Epidermis, ducts	Mucilage	Leaf (blade)	11
Ziziphus apetala	Epidermis, ducts	Mucilage	Leaf (blade)	11
Ziziphus calophylla	Ducts	Mucilage	Leaf (blade), stem	11, 12
Ziziphus cotinifolia	Idioblasts, ducts	Mucilage, tannins	Leaf (blade)	11

Ziziphus elegans	Epidermis, ducts	Mucilage	Leaf (blade)	11
Ziziphus funiculosa	Epidermis, ducts	Mucilage	Leaf (blade)	11
Ziziphus glabrata	Idioblasts, cavities, ducts	Mucilage, tannins	Leaf (blade, petiole)	11, 20
Ziziphus horsfieldii	Epidermis, ducts	Mucilage	Leaf (blade)	11
Ziziphus incurva	Ducts	Mucilage	Leaf (blade)	11
Ziziphus javanensis	Epidermis, ducts	Mucilage	Leaf (blade)	11
Ziziphus jujuba	Epidermis, idioblasts, cavities, ducts, osmophores	Mucilage, tannins, terpenes	Flower, leaf (blade, petiole), stem	11, 17, 20, 21, 24
Ziziphus linnaei	Epidermis, ducts	Mucilage	Leaf (blade)	11
Ziziphus lotus	Epidermis, ducts	Mucilage	Flower, leaf (blade), stem	11, 81
Ziziphus mauritiana	Epidermis, ducts	Mucilage, tannins	Leaf (blade)	11
Ziziphus mistol	Idioblasts, osmophores	Mucilage, terpenes	Flower	24
Ziziphus mucronata	Epidermis, idioblasts, ducts, osmophores	Mucilage, terpenes	Flower, leaf (blade)	11, 24
Ziziphus nummularia	Epidermis, idioblasts, cavities, ducts	Mucilage, tannins	Leaf (blade, petiole), stem	11, 17, 20
Ziziphus oenopolia	Epidermis, idioblasts, cavities, ducts	Mucilage, tannins	Leaf (blade, petiole)	11, 12, 20
Ziziphus oxyphylla	Epidermis, ducts	Mucilage	Leaf (blade)	11

	Ziziphus rugosa	Epidermis, idioblasts, cavities, ducts	Mucilage, tannins	Leaf (blade, petiole)	11, 20
	Ziziphus spina-christi	Epidermis, idioblasts, ducts	Mucilage, tannins	Leaf (blade)	11
	Ziziphus trinervis	Epidermis, ducts	Mucilage	Leaf (blade)	11
	Ziziphus xylopyrus	Epidermis, idioblasts, cavities, ducts	Mucilage, tannins	Leaf (blade, petiole)	11, 20
	Ziziphus zeyheriana	Epidermis, ducts	Mucilage	Leaf (blade)	11
Phyliceae	*Noltea africana*	Gladular leaf-teeth, idioblasts	Tannins	Leaf (blade)	10, 12, 13
	Phylica cylindrica	Idioblasts	Tannins	Leaf (blade)	10
	Phylica nitida	Gladular leaf-teeth	?	Leaf (blade)	12, 13
	Phylica pinea	Idioblasts	Tannins	Leaf (blade)	10
	Phylica pubescens	Idioblasts	Tannins	Leaf (blade)	10
	Phylica rigida	Idioblasts	Tannins	Leaf (blade)	10
	Phylica rosmarinifolia	Idioblasts	Tannins	Leaf (blade)	10
Pomaderreae	*Cryptandra sp.*	Epidermis, idioblasts	Mucilage	Leaf (blade)	12, 13
	Cryptandra amara	Epidermis, idioblasts	Mucilage, tannins	Leaf (blade)	10
	Cryptandra arbutiflora	Epidermis	Mucilage	Leaf (blade)	10

	Cryptandra arbutiflora var. tubulosa	Epidermis, idioblasts	Mucilage, tannins	Leaf (blade)	10
	Cryptandra australis	Epidermis, idioblasts	Mucilage, tannins	Leaf (blade)	10
	Cryptandra leucopogon	Epidermis, idioblasts	Mucilage, tannins	Leaf (blade)	10
	Cryptandra mutila	Epidermis, idioblasts	Mucilage, tannins	Leaf (blade)	10
	Cryptandra parvifolia	Epidermis, idioblasts	Mucilage, tannins	Leaf (blade)	10
	Cryptandra scoparia	Epidermis, idioblasts	Mucilage, tannins	Leaf (blade)	10
	Cryptandra spinescens	Epidermis, idioblasts	Mucilage, tannins	Leaf (blade)	10
	Cryptandra tomentosa	Epidermis, idioblasts	Mucilage, tannins	Leaf (blade)	10
	Pomaderris sp.	Epidermis, idioblasts	Mucilage, tannins	Leaf (blade)	12, 13
	Pomaderris andromedifolia	Epidermis, idioblasts	Mucilage, tannins	Leaf (blade)	11, 12
	Pomaderris apetala	Epidermis, idioblasts	Mucilage, tannins	Leaf (blade, petiole)	10, 20
	Pomaderris discolor	Epidermis, idioblasts	Mucilage, tannins	Leaf (blade)	10, 12
	Pomaderris elliptica	Epidermis, idioblasts	Mucilage, tannins	Leaf (blade)	10
	Pomaderris lanigera	Epidermis, idioblasts	Mucilage, tannins	Leaf (blade)	10, 12
	Pomaderris ligustrina	Idioblasts	Tannins	Leaf (blade)	10
	Pomaderris phylicifolia	Epidermis, idioblasts	Mucilage, tannins	Leaf (blade)	10

	Pomaderris prunifolia	Epidermis, idioblasts	Mucilage, tannins	Leaf (blade)	10
	Pomaderris racemosa	Epidermis, idioblasts	Mucilage, tannins	Leaf (blade)	10
	Pomaderris wendlandiana	Idioblasts	Tannins	Leaf (blade)	10, 12
	Spyridium coactilifolium	Epidermis, idioblasts	Mucilage, tannins	Leaf (blade)	10
	Spyridium eriocephalum	Epidermis, idioblasts	Mucilage, tannins	Leaf (blade)	10
	Spyridium globulosum	Idioblasts	Tannins	Leaf (blade)	10
	Spyridium gunnii	Idioblasts	Tannins	Leaf (blade)	10
	Spyridium majoranifolium	Epidermis, idioblasts	Mucilage, tannins	Leaf (blade)	10
	Spyridium obcordatum	Epidermis, idioblasts	Mucilage, tannins	Leaf (blade)	10
	Spyridium obovatum	Epidermis, idioblasts	Mucilage, tannins	Leaf (blade)	10, 12
	Spyridium ulicinum	Idioblasts	Tannins	Leaf (blade)	10
	Spyridium vexilliferum	Idioblasts	Tannins	Leaf (blade)	10
	Trymalium sp.	Epidermis, idioblasts	Mucilage	Leaf (blade)	12, 13
	Trymalium ledifolium	Idioblasts	Tannins	Leaf (blade)	10
	Trymalium odoratissimum	Idioblasts	Tannins	Leaf (blade)	10
Rhamneae	*Auerodendron reticulatum*	Idioblasts, cavities, ducts	Mucilage, tannins, resin	Leaf (blade)	11, 12

Auerodendron northropianum	Idioblasts, ducts	Mucilage, tannins	Leaf (blade)	11
Berchemia sp.	Epidermis, idioblasts	Mucilage, tannins	Leaf (blade, petiole)	12, 13
Berchemia discolor	Epidemis, idioblasts	Mucilage, tannins	Leaf (blade)	11, 12
Berchemia floribunda	Epidermis, idioblasts	Mucilage, tannins	Leaf (blade)	11
Berchemia lineata	Epidermis, idioblasts	Mucilage, tannins	Leaf (blade)	11
Berchemia scandens	Epidermis, idioblasts	Mucilage, tannins	Leaf (blade)	11
Condalia sp.	Epidermis, idioblasts, cavities, ducts	Mucilage, tannins	Leaf (blade, petiole)	12, 13, 15
Condalia buxifolia	Idioblasts, osmophores	Mucilage, terpenes	Flower	24
Condalia ericoides	Hypodermis	Mucilage	Leaf (blade)	11, 12
Condalia hookeri	Idioblasts, ducts	Mucilage, tannins	Leaf (blade)	11
Condalia mexicana	Hypodermis, idioblasts, ducts	Mucilage, tannins	Leaf (blade)	11, 12
Condalia microphylla	Hypodermis, idioblasts	Mucilage, tannins	Leaf (blade)	11, 12
Frangula sp.	Ducts	Mucilage	Leaf (blade), stem	12
Frangula alnus	Epidermis, idioblasts, cavities, ducts,	Mucilage, tannins	Flower, fruit, leaf (blade, petiole), stem	10, 11, 12, 13, 19, 82, 83, 84, 85
Frangula azorica	Epidermis, idioblasts, cavities, ducts	Mucilage, tannins	Leaf (blade), stem	11, 86

Frangula californica	Epidermis, idioblasts, ducts	Mucilage, tannins	Flower, fruit, leaf, stem	11, 13, 82
Frangula capreifolia	Epidermis, idioblasts, ducts	Mucilage, tannins	Leaf (blade)	11
Frangula caroliniana	Epidermis, idioblasts, ducts	Mucilage, tannins	Leaf (blade)	11
Frangula crenata	Epidermis, idioblasts, ducts	Mucilage, tannins	Leaf (blade)	11
Frangula grandifolia	Epidermis, idioblasts, ducts	Mucilage, tannins	Leaf (blade)	11
Frangula microphylla	Epidermis, idioblasts, ducts	Mucilage, tannins	Leaf (blade)	11
Frangula palmeri	Epidermis, idioblasts, ducts	Mucilage, tannins	Leaf (blade)	11
Frangula purshiana	Epidermis, idioblasts, ducts	Mucilage, tannins	Leaf (blade), stem	11, 13, 83
Frangula rupestris	Epidermis, idioblasts, cavities, ducts	Mucilage, flavonoids, tannins	Leaf (blade, petiole), stem	10, 11, 22
Frangula sphaerosperma	Epidermis, idioblasts, ducts	Mucilage, tannins	Leaf (blade)	11
Karwinskia sp.	Cavities, ducts	Mucilage, resin	Leaf (petiole)	12, 13
Karwinskia humboldtiana	Cavities, ducts	Mucilage, resin	Leaf (blade)	11, 12
Karwinskia sessilifolia	Cavities, ducts	Mucilage, resin	Leaf (blade)	11
Krugiodendron sp.	Idioblasts	Tannins	Leaf (blade)	12, 13

Krugiodendron ferreum	Epidermis, idioblasts	Tannins	Leaf (blade)	11
Pseudoziziphus parryi	Epidermis, idioblasts	Mucilage, tannins	Leaf (blade)	11
Reynosia sp.	Epidermis, idioblasts	Mucilage	Leaf (blade)	12, 13
Reynosia barbatula	Idioblasts	Mucilage	Flower	23
Reynosia domingensis	Idioblasts	Mucilage	Flower	23
Reynosia guama	Idioblasts	Mucilage	Flower	23
Reynosia jamaicensis	Idioblasts	Mucilage	Flower	23
Reynosia krugii	Idioblasts	Mucilage	Flower	23
Reynosia moaensis	Idioblasts	Mucilage	Flower	23
Reynosia regia	Idioblasts	Mucilage	Flower	23
Reynosia retusa	Idioblasts	Tannins	Leaf (blade)	11
Reynosia revoluta	Hypodermis, idioblasts, ducts	Mucilage, resin	Flower, leaf (blade)	11, 12, 23
Reynosia uncinata	Idioblasts	Mucilage	Flower	23
Reynosia wrightii	Epidermis, idioblasts	Mucilage, tannins	Flower, leaf (blade)	11, 23
Rhamnella sp.	Epidermis, idioblasts, cavities, ducts	Mucilage, tannins	Leaf (blade, petiole)	12, 13, 15
Rhamnella franguloides	Epidermis, idioblasts	Mucilage, tannins	Leaf (blade)	11, 12

	Rhamnella vitiensis	Epidermis, idioblasts, ducts	Mucilage, tannins	Leaf (blade)	11
	Rhamnidium sp.	Epidermis, idioblasts, cavities, ducts	Mucilage	Leaf (blade, petiole)	12, 13
	Rhamnidium elaeocarpum	Epidermis, idioblasts, cavities, ducts and colleters	Mucilage, phenols, terpens, lipids, resin, tannins	Flower, leaf (blade)	Present study, 11
	Rhamnidium glabrum	Epidermis, cavities, ducts	Mucilage, resin, tannins	Leaf (blade)	11
	Rhamnus sp.	Glandular leaf-teeth, idioblasts	Mucilage, tannins	Leaf (blade, petiole)	12, 13, 15
	Rhamnus alaternus	Idioblasts	Tannins	Leaf (blade)	10, 11
	Rhamnus alnifolia	Idioblasts	Tannins	Leaf (blade)	11
	Rhamnus alpina	Glandular leaf-teath, idioblasts	Tannins	Leaf (blade)	11, 12
	Rhamnus cathartica	Idioblasts, osmophores	Tannins, terpenes	Flower, leaf (blade)	10, 12, 24
	Rhamnus cornifolia	Idioblasts	Tannins	Leaf (blade)	11
	Rhamnus costata	Idioblasts	Tannins	Leaf (blade)	11
	Rhamnus crocea	Idioblasts	Tannins	Leaf (blade)	11
	Rhamnus dauurica	Idioblasts	Tannins	Leaf (blade)	11
	Rhamnus diffusa	Epidermis, ducts	Mucilage, tannins	Leaf (blade)	11
	Rhamnus erythroxylon	Idioblasts	Tannins	Leaf (blade)	11

	Rhamnus infectoria	Idioblasts	Tannins	Leaf (blade)	11, 12
	Rhamnus × intermedia	Idioblasts	Tannins	Leaf (blade)	11
	Rhamnus japonica	Idioblasts	Tannins	Leaf (blade)	11
	Rhamnus lanceolata	Idioblasts	Tannins	Leaf (blade)	11
	Rhamnus longifolia	Idioblasts	Tannins	Leaf (blade)	11
	Rhamnus lycioides subsp. oleoides	Idioblasts	Tannins	Leaf (blade)	10, 11
	Rhamnus lycioides subsp. graeca	Idioblasts	Tannins	Leaf (blade)	10, 11
	Rhamnus napalensis	Idioblasts	Tannins	Leaf (blade)	11
	Rhamnus oleoides	Idioblasts	Tannins	Leaf (blade)	11
	Rhamnus orbiculata	Idioblasts	Tannins	Leaf (blade)	11
	Rhamnus parvifolia	Idioblasts	Tannins	Leaf (blade)	11
	Rhamnus prinoides	Idioblasts	Tannins	Leaf (blade)	10, 11
	Rhamnus pumila	Idioblasts	Tannins	Leaf (blade)	10, 11
	Rhamnus purpurea	Idioblasts	Tannins	Leaf (blade)	11
	Rhamnus rhodopea	Idioblasts	Tannins	Leaf (blade)	11
	Rhamnus saxatilis	Idioblasts	Tannins	Leaf (blade)	10, 11

Rhamnus serrata	Idioblasts	Tannins	Leaf (blade)	11
Rhamnus smithii	Idioblasts	Tannins	Leaf (blade)	11
Rhamnus × spathulifolia	Idioblasts	Tannins	Leaf (blade)	11
Rhamnus staddo	Idioblasts	Tannins	Leaf (blade)	11
Rhamnus triquetra	Idioblasts	Tannins	Leaf (blade)	11
Rhamnus wightii	Idioblasts, cavities	Tannins	Leaf (blade, petiole)	11, 20
Sageretia sp.	Epidermis, idioblasts, ducts	Mucilage	Leaf (blade)	12, 13
Sageretia brandrethiana	Epidermis, idioblasts, ducts	Mucilage, tannins	Leaf (blade)	10
Sageretia elegans	Epidermis, idioblasts, ducts	Mucilage, tannins	Leaf (blade)	10
Sageretia filiformis	Idioblasts, ducts	Mucilage, tannins	Leaf (blade)	10
Sageretia hamosa	Idioblasts, ducts	Mucilage, tannins	Leaf (blade)	10
Sageretia minutiflora	Epidermis, idioblasts, ducts	Mucilage, tannins	Leaf (blade)	10
Sageretia thea	Glandular leaf-teath, epidermis, idioblasts, ducts	Mucilage, tannins	Leaf (blade)	10, 11
Sageretia wrightii	Idioblasts	Tannins	Leaf (blade)	10
Scutia sp.	Epidermis, idioblasts, ducts	Mucilage, tannins	Leaf (blade)	12, 13

	Scutia buxifolia	Osmophores	Terpenes	Flower	24
	Scutia myrtina	Epidermis, idioblasts, ducts	Mucilage, tannins	Leaf (blade, petiole)	10, 12, 20
Ventilagineae	*Smythea bombaiensis*	Epidermis, idioblasts	Mucilage, tannins	Leaf (blade, petiole)	11, 20
	Smythea calpicarpa	Idioblasts	Tannins	Leaf (blade)	11
	Smythea lanceata	Idioblasts	Tannins	Leaf (blade)	11
	Smythea macrocarpa	Idioblasts	Tannins	Leaf (blade)	11
	Ventilago sp.	Epidermis, idioblasts, cavities, ducts	Mucilage	Leaf (blade, petiole)	12, 13
	Ventilago denticulata	Idioblasts	Tannins	Leaf (petiole)	20
	Ventilago leiocarpa	Epidermis, idioblasts, ducts	Mucilage, taninns	Leaf (petiole)	11
	Ventilago madraspatana	Epidermis, idioblasts, ducts	Mucilage, taninns	Leaf (blade), stem	11, 12

ReferenceReferences

1. The Angiosperm Phylogeny Group; Chase, M.W.; Christenhusz, M.J.M.; Fay, M.F.; Byng, J.W.; Judd, W.S.; Soltis, D.E.; Mabberley, D.J.; Sennikov, A.N.; Soltis, P.S.; Stevens, P.F. An update of the Angiosperm Phylogeny Group classification for the orders and families of flowering plants: APG IV. *Bot. J. Linn. Soc.* **2016**, *181*, 1–20.
2. Rhamnaceae in Flora do Brasil 2020 em Construção. Jardim Botânico do Rio de Janeiro. Available online: http://floradobrasil.jbrj.gov.br/reflora/floradobrasil/FB207 (accessed on 9 October 2020).
3. Medan, D. Reproductive phenology, pollination biology, and gynoecium development in *Discaria americana* (Rhamnaceae). *N. Z. J. Bot.* **1991**, *29*, 31–42.
4. Medan, D.; D'Ambrogio, A.C. Reproductive biology of the andromonoecious shrub *Trevoa quinquenervia* (Rhamnaceae). *Bot. J. Linn. Soc.* **1998**, *126*, 191–206.
5. Medan, D.; Arce, M.E. Reproductive biology of the Andean-disjunct genus *Retanilla* (Rhamnaceae). *Plant Syst. Evol.* **1999**, *218*, 281–298.
6. Medan, D.; Toretta, J.P. The reproduction of *Colletia hystrix* and late flowering in *Colletia* (Rhamnaceae: Colletieae). *Plant Syst. Evol.* **2014**, *301*, 1181–1189.
7. Cerino, M.C.; Richard, G.A.; Torretta, J.P.; Gutierrez, H.F.; Pensiero, J.F. Reproductive biology of *Ziziphus mistol* Griseb. (Rhamnaceae), a wild fruit tree of saline environments. *Flora Morphol. Distrib. Funct. Ecol. Plants* **2015**, *211*, 18–25.
8. Medan, D.; Aagesen, L. Comparative flower and fruit structure in the Colletieae (Rhamnaceae). *Bot. Jahrb. Syst.* **1995**, *117*, 531–564.
9. Gotelli, M.M.; Galati, B.G.; Medan, D. Morphological and ultrastructural studies of floral nectaries in Rhamnaceae. *J. Torrey Bot. Soc.* **2017**, *144*, 63–73.

10. Gemoll, K. Anatomisch-systematische untersuchung des blattes der Rhamneen aus den triben: Rhamneen, Colletieen und Gouanieen. *Beihefte Botanischen Centralblatt* **1902**, *12*, 351–424.
11. Herzog, T.H. Anatomisch-systematische untersuchung des blattes der Rhamneen aus den triben: Ventilagineen, Zizypheen und Rhamneen. *Beihefte Botanischen Centralblatt* **1903**, *15*, 95–207.
12. Solereder, H. *Systematic Anatomy of the Dicotyledons: A Handbook for Laboratories of Pure and Applied Botany*; Clarendon Press: Oxford, UK, 1908; Volumes 1 and 2, pp. 218–221, 885–889.
13. Metcalfe, C.R.; Chalk, L. *Anatomy of the Dicotyledons: Leaves, Stem and Wood in Relation to Taxonomy with Notes on Economic Uses*; Clarendon Press: Oxford, England, 1957; Volume 1, pp. 404–413.
14. Medan, D. Anatomia foliar de *Kentrothamnus* (Rhamnaceae). *Bol. Soc. Argent. Bot.* **1974**, *16*, 83–88.
15. Gregory, M.; Baas, P. A survey of mucilage cells in vegetative organs of the dicotyledons. *Isr. J. Plant Sci.* **1989**, *38*, 125–174.
16. Mantese, A.; Medan, D. Anatomia y arquitectura foliares de *Retanilla* (Rhamnaceae). *Darwiniana* **1992**, *31*, 253–259.
17. Clifford, S.C.; Arndt, S.K.; Popp, M.; Jones, H.G. Mucilages and polysaccharides in *Ziziphus* species (Rhamnaceae): Localization, composition and physiological roles during drought-stress. *J. Exp. Bot.* **2002**, *53*, 131–138.
18. Medan, D.; Schirarend, C. Rhamnaceae. In *Flowering Plants: Dicotyledons*; Kubitzki, K., Ed.; Springer-Verlag: Berlin/Heidelberg, Germany, 2004; pp. 320–338.
19. Serdar, B.; Çoskunçelebi, K.; Terzioglu, S.; Hampe, A. Anatomical notes on Turkish *Frangula alnus* Mill. (Rhamnaceae). *Plant Biosyst.* **2007**, *141*, 69–74.
20. Shisode, S.B.; Patil, D.A. Petiolar anatomy in some Rhamnaceae. *Curr. Bot.* **2011**, *2*, 22–25.
21. Sivasankari, M.P.; Sankaravadivoo, A. Leaf anatomy of *Ziziphus mauritiana* Lam. *Int. J. Curr. Res. Biosci. Plant Biol.* **2017**, *4*, 73–79.
22. Arsenijević, J.; Drobac, M.; Slavkovska, V.; Kovačević, N.; Lakušić, B. Anatomical analysis and phytochemical screening of *Frangula rupestris* (Scop.) Schur (Rhamnaceae). *Bot. Serb.* **2018**, *42*, 231–239.
23. Schirarend, C.; Hoffmann, P. Untersuchungen zur blütenmorphologie der gattung *Reynosia* Griseb. (Rhamnaceae). *Flora* **1993**, *188*, 275–286.
24. Gotelli, M.M.; Galati, B.G.; Zarlavsky, G.; Nicolau, M.; Reposi, S. Localization, morphology, anatomy and ultrastructure of osmophores in species of Rhamnaceae. *Protoplasma* **2020**, *257*, 1109–1121.
25. Asgarpanah, J.; Haghighat, E. Phytochemistry and pharmacologic properties of *Ziziphus spina christi* (L.) Willd. *Afr. J. Pharm. Pharmacol.* **2012**, *6*, 2332–2339.
26. Abdel-Zaher, A.O.; Salim, S.Y.; Assaf, M.H.; Abdel-Hady, R.H. Antidiabetic activity and toxicity of *Zizyphus spina-christi* leaves. *J. Ethnopharmacol.* **2005**, *101*, 129–138.
27. Pawlowska, A.M.; Camangi, F.; Bader, A.; Braca, A. Flavonoids of *Zizyphus jujuba* L. and *Zizyphus spina-christi* (L.) Willd (Rhamnaceae) fruits. *Food Chem.* **2009**, *112*, 858–862.
28. Hyun, T.K.; Eom, S.H.; Yu, C.Y.; Roitsch, T. *Hovenia dulcis*—An Asian traditional herb. *Planta Med.* **2010**, *76*, 943–949.
29. Abalaka, M.E.; Mann, A.; Adeyemo, S.O. Studies on in-vitro antioxidant and free radical scavenging potential and phytochemical screening of leaves of *Ziziphus mauritiana* L. and *Ziziphus spina-christi* L. compared with ascorbic acid. *J. Med. Genet. Genom.* **2011**, *3*, 28–34.
30. Wang, M.; Jiang, C.; Ma, L.; Zhang, Z.; Cao, L.; Liu, J.; Zeng, X. Preparation, preliminary characterization and immunostimulatory activity of polysaccharide fractions from the peduncles of *Hovenia dulcis*. *Food Chem.* **2013**, *138*, 41–47.
31. Ashraf, A.; Sarfrazi, R.A.; Anwar, F.; Shahid, S.A.; Alkharfy, K.M. Chemical composition and biological activities of leaves of *Ziziphus mauritiana* L. native to Pakistan. *Pak. J. Bot.* **2015**, *47*, 367–376.
32. Brito, S.M.O.; Coutinho, H.D.M.; Talvani, A.; Coronel, C.; Barbosa, A.G.R.; Vega, C.; Figueredo, F.; Tintino, S.; Lima, L.F.; Boligon, A.A.; et al. Analysis of bioactivities and chemical composition of *Ziziphus joazeiro* Mart. using HPLC–DAD. *Food Chem.* **2015**, *186*, 185–191.
33. Ji, X.; Peng, Q.; Yuan, Y.; Shen, J.; Xie, X.; Wang, M. Isolation, structures and bioactivities of the polysaccharides from jujube fruit (*Ziziphus jujuba* Mill.): A review. *Food Chem.* **2017**, *227*, 349–357.
34. Hussein, A.S. *Ziziphus spina-christi*: Analysis of bioactivities and chemical composition. In *Wild Fruits: Composition, Nutritional Value and Products*; Mariod, A.A., Ed.; Springer: Cham, Switzerland, 2019; pp. 175–197.
35. Yang, B.; Wu, Q.; Luo, Y.; Yang, Q.; Chen, G.; Wei, X.; Kan, J. Japanese grape (*Hovenia dulcis*) polysaccharides: New insight into extraction, characterization, rheological properties, and bioactivities. *Int. J. Biol. Macromol.* **2019**, *134*, 631–644.
36. Mahajan, R.T.; Chopda, M.Z. Phyto-pharmacology of *Ziziphus jujuba* Mill—A plant review. *Pharmacogn. Rev.* **2009**, *3*, 320–329.

37. Silva, L.A.; Sales, J.F.; Guimarães, R.M.; Oliveira, J.A.; Vasconcelos Filho, S.C. Morphological aspects of fruits, seeds and seedlings of *Rhamnidium elaeocarpum* Reissek. *Semin. Cienc. Agrar.* **2015**, *36*, 1179–1190.
38. Fahn, A. *Secretory Tissues in Plants*; Academic Press: London, UK, 1979.
39. Evert, R.F. *Esau's Plant Anatomy—Meristems, Cells, and Tissues of the Plant Body: Their Structure, Function, and Development*, 3rd ed.; John Wiley & Sons Inc: Hoboken, NJ, USA, 2006.
40. Rudall, P.J. Laticifers in Euphorbiaceae—A conspectus. *Bot. J. Linn. Soc.* **1987**, *94*, 143–163.
41. Marinho, C.R.; Teixeira, S.P. Novel reports of laticifers in Moraceae and Urticaceae: Revisiting synapomorphies. *Plant Syst. Evol.* **2019**, *305*, 13–31.
42. Demarco, D. Floral glands in asclepiads: Structure, diversity and evolution. *Acta Bot. Bras.* **2017**, *31*, 477–502.
43. Marin, M.; Koko, V.; Duletić-Lašević, S.; Marin, P.D.; Rančić, D.; Dajic-Stevanovic, Z. Glandular trichomes on the leaves of *Rosmarinus officinalis*: Morphology, stereology and histochemistry. *S. Afr. J. Bot.* **2006**, *72*, 378–382.
44. Ciccarelli, D.; Garbari, F.; Pagni, A.M. The flower of *Myrtus communis* (Myrtaceae): Secretory structures, unicellular papillae, and their ecological role. *Flora* **2008**, *203*, 85–93.
45. Hauenschild, F.; Matuszak, S.; Muellner-Riehl, A.N.; Favre, A. Phylogenetic relationships within the cosmopolitan buckthorn family (Rhamnaceae) support the resurrection of *Sarcomphalus* and the description of *Pseudoziziphus* gen. nov. *TAXON* **2016**, *65*, 47–64.
46. Thomas, V. Structural, functional and phylogenetic aspects of the colleter. *Ann. Bot.* **1991**, *68*, 287–305.
47. Machado, A.F.P.; Souza, A.M.; Leitão, C.A.E. Secretory structures at syconia and flowers of *Ficus enormis* (Moraceae): A specialization at ostiolar bracts and the first report of inflorescence colleters. *Flora* **2013**, *208*, 45–51.
48. Foster, A.S. Plant idioblasts: Remarkable examples of cell specialization. *Protoplasma* **1956**, *46*, 184–193.
49. Schultz, J.C. Tannin-insect interactions. In *Chemistry and Significance of Condensed Tannins*; Hemingway, R.W., Karehesy, J.J., Eds.; Plenum Press: New York, NY, USA, 1989; pp. 417–433.
50. Peters, N.K.; Verma, D.P.S. Phenolic compounds as regulators of gene expression in plant-microbe interactions. *Mol. Plant Microbe Interact.* **1990**, *3*, 4–8.
51. Schlesinger, W.H. *Biogeochemistry: An Analysis of Global Change*; Academic Press: San Diego, CA, USA, 1991.
52. Appel, H.M. Phenolics in ecological interactions: The importance of oxidation. *J. Chem. Ecol.* **1993**, *19*, 1521–1552.
53. Castro, M.M.; Demarco, D. Phenolic compounds produced by secretory structures in plants: A brief review. *Nat. Prod. Commun.* **2008**, *3*, 1273–1284.
54. Leme, F.M.; Borella, P.H.; Marinho, C.R.; Teixeira, S.P. Expanding the laticifer knowledge in Cannabaceae: Distribution, morphology, origin, and latex composition. *Protoplasma* **2020**, *257*, 1183–1199.
55. Guignard, L.; Colin, H. Sur la présence de réservoirs a gomme chez les Rhamnées. *Bull. Soc. Bot. France* **1888**, *35*, 325–327.
56. Bouchet, P. Étude ultrastructurale des cellules constituant les poches "lysigènes" à mucilage de la bourdaine: *Rhamnus frangula* L. *C. R. Hebd. Séances Acad. Sci.* **1974**, *279*, 1073–1076.
57. Pool, A. New species, combinations, and lectotypifications in Neotropical and northern Mexican *Frangula* (Rhamnaceae). *Novon* **2013**, *22*, 447–467.
58. Wollenweber, E.; Dörr, M.; Bohm, B.A.; Roitman, J.N. Exudate flavonoids of eight species of *Ceanothus* (Rhamnaceae). *Z. Naturforsch.* **2004**, *59*, 459–462.
59. Sun, M.; Naeem, R.; Su, J.-X.; Cao, Z.-Y.; Burleigh, J.G.; Soltis, P.S.; Soltis, D.E.; Chen, Z-D. Phylogeny of the Rosidae: A dense taxon sampling analysis. *J. Syst. Evol.* **2016**, *54*, 363–391.
60. Duffey, S.S.; Stout, M.J. Antinutritive and toxic components of plant defense against insects. *Arch. Insect Biochem. Physiol.* **1996**, *32*, 3–37.
61. Romeo, J.T.; Saunders, J.A.; Barbosa, P. *Phytochemical Diversity and Redundancy in Ecological Interactions*; Plenum Press: New York, NY, USA, 1996.
62. Agrawal, A.A.; Fishbein, M. Plant defense syndromes. *Ecology* **2006**, *87*, S132–S149.
63. POWO. Plants of the World Online. Facilitated by the Royal Botanic Gardens, Kew. 2019. Available online: http://www.plantsoftheworldonline.org/ (accessed on 10 November 2020).
64. Clark, G. *Staining Procedures*, 4th ed.; Williams & Wilkins: Baltimore, MD, USA, 1981.
65. Gerrits, P.O.; Horobin, R.W. *The Application of Glycol Methacrylate in Histotechnology; Some Fundamental Principles*; Faculteit der Geneeskunde, Rijksuniversiteit Groningen: Groningen, The Netherlands, 1991.
66. O'Brien, T.P.; Feder, N.; McCully, M.E. Polychromatic staining of plant cell walls by toluidine blue O. *Protoplasma* **1964**, *59*, 368–373.
67. Jensen, W.E. *Botanical Histochemistry: Principles and Practice*; W.H. Freeman & Co: San Francisco, CA, USA, 1962.
68. Pearse, A.G.E. *Histochemistry: Theoretical and Applied*. C. Livingstone: Edinburgh, UK, 1972.

69. David, R.; Carde, J. Coloration différentielle des inclusions lipidiques et terpeniques des pseudophylles du pin maritime au moyen du reactif Nadi. *C. R. Hebd. Seanc. Acad. Sci. Paris* **1964**, *258*, 1338–1340.
70. Karnovsky, M.J. A formaldehyde-glutaraldehyde fixative of high osmolarity for use in eletron microscopy. *J. Cell Biol.* **1965**, *27*, 137–138.
71. Matthews, M.L.; Endress, P.K. Floral structure and systematics in four orders of rosids, including a broad survey of floral mucilage cells. *Plant Syst. Evol.* **2006**, *260*, 199–221.
72. Holm, T. *Ceanothus americanus* L. and *ovatus* Desf.; a morphological and anatomical study. *Am. J. Sci.* **1906**, *22*, 523–530.
73. Mantese, A.; Medan, D. Anatomia y arquitectura foliares de *Colletia* y *Adolphia* (Rhamnaceae). *Darwiniana* **1993**, *32*, 91–97.
74. Medan, D.; Mantese, A. Contribución a la anatomía y arquitectura foliares de *Talguenea* (Rhamnaceae). *Kurtziana* **1989**, *20*, 95–100.
75. Buono, R.A.; Oliveira, A.B.; Paiva, E.A.S. Anatomy, ultrastructure and chemical composition of food bodies of *Hovenia dulcis* (Rhamnaceae). *Ann. Bot.* **2008**, *101*, 1341–1348.
76. Shahbaz, S.E.; Shareef, N.M. Use of morphological and anatomical characters to delimit varieties of *Paliurus spina-christi* Mill. (Rhamnaceae). *Innovaciencia* **2018**, *6*, 1–14.
77. Nascimento, A.M.; Torres, J.C.; Marques, C.A. Caracterização morfo-anatômica e testes fitoquímicos em amostras comerciais de *Ziziphus joazeiro* Mart. (Rhamnaceae). *Rev. Fitos* **2016**, *10*, 375–547.
78. Colares, M.N.; Arambarri, A.M. *Ziziphus mistol* (Rhamnaceae): Morfo-anatomía y arquitectura foliar. *Lat. Am. J. Pharm.* **2008**, *27*, 568–577.
79. Nasri-Ayachi, M.B.; Nabli, M.A. Floral biology study of *Ziziphus lotus* L. *Acta Hortic.* **2009**, *840*, 337–342.
80. Hasler, O. Entwicklungsgeschichte und Vergleichende Anatomie der Pharmakognostisch Wichtigen Rhamnusrinden Unter Besonderer Berücksichtigung der Calciumoxalat-Bildung. Ph.D. Thesis, ETH Zürich, Zürich, Switzerland, 1936.
81. Lacombe, N.R.; Youngken, H.W. Studies on the anatomy of *Rhamnus lanceolata* Pursh and *Rhamnus frangula* L. *J. Am. Pharm. Assoc.* **1943**, *32*, 193–202.
82. Malmir, M.; Curica, C.; Gomes, E.T.; Serrano, R.; Silva, O. Contribution of light and electron microscopy to identification of bark from *Frangula azorica*, an Azorean medicinal plant. *Microsc. Microanal.* **2015**, *21*, 1296–1303.
